# Yellow Fever in a Worker Returning to China from Angola, March 2016

**DOI:** 10.3201/eid2207.160469

**Published:** 2016-07

**Authors:** Yun Ling, Jun Chen, Qin Huang, Yunwen Hu, Aihong Zhu, Shanke Ye, Lie Xu, Hongzhou Lu

**Affiliations:** Shanghai Public Health Clinical Center, Shanghai, China

**Keywords:** yellow fever, China, Angola, vaccine, flavivirus, yellow fever virus, viruses

**To the Editor:** Yellow fever is disease caused by a flavivirus that is transmitted to humans and nonhuman primates through the bites of infected mosquitoes. In 2013, an estimated 130,000 persons in Africa experienced fever with jaundice or hemorrhage associated with yellow fever; ≈78,000 of these infections were fatal (*1*).

Recently, an outbreak of yellow fever was reported in Angola (*2*). This serious viral disease affects persons living in and visiting tropical regions of Africa and Central and South America (*3*). No case of yellow fever had been confirmed in China until this year (*3*). With the increased population movement between Africa and China, the risk for yellow fever in China is increasing.

In March 2016, a 34-year-old man who had recently returned to China from Angola sought medical treatment at the Shanghai Public Health Clinical Center in Shanghai, China. He reported a 4-day history of malaise, myalgia, weakness, nausea, vomiting, and fever reaching 38.8°C. The patient had been treated with several antimicrobial drugs when he was in Angola, but symptoms did not resolve. He had no history of immunodeficiency or immune-inhibitory drug use. No endocrine, metabolic, or autoimmune abnormalities were found.

Nine years earlier, the patient had undergone cardiac valve replacement for rheumatoid heart disease and was currently receiving warfarin therapy. Because his treating physicians were concerned about the potential effect of yellow fever vaccine on the patient’s international normalized ratio (ratio of reference to measured prothrombin times), the patient traveled to Africa for work without receiving vaccination for yellow fever.

Physical examination revealed a temperature of 37°C. Neither rash nor jaundice were evident. Blood examination revealed a low leukocyte count (1.66 × 10^9^ cells/L [reference range 3.50–9.50 × 10^9^ cells/L]), a low absolute lymphocyte count (0.92 × 10^9^ cells/L [1.1–3.2 × 10^9^ cells/L), a normal erythrocyte count (4.60 × 10^12^ cells/L [4.30–5.80 × 10^12^ cells/L]), and a low platelet count (43 × 10^9^ platelets/L [125–350 × 10^9^ platelets/L). The patient had low levels of circulating CD3+ cells (540/μL [690–2,540/μL) and CD8+ cells (97/μL [190–1,140/μL]) and normal levels of CD4+ T-cells.

C-reactive protein level was 4.31 mg/L (reference range 0–3.0 mg/L), lactate dehydrogenase was 1,086 U/L (109–245 U/L), alanine aminotransferase was 882 U/L (7–40 U/L), total bilirubin was 13.5 μmol/L (0–17 μmol/L), and direct bilirubin was 7.4 μmol/L (0–5.4 μmol/L). The patient had normal levels of thyroid-stimulating hormone, and no DNA, nuclear, or thyroglobulin antibodies were detected.

Test results for HIV, malaria, and dengue virus infection were negative. Serum and urine samples were positive for yellow fever virus and negative for dengue and Zika viruses by PCR. These results were confirmed by the Shanghai Center for Disease Control and Prevention and the China Center for Disease Control and Prevention. Yellow fever virus RNA remained detectable 9 days after symptom onset in serum and for an additional 3 days in urine and feces. 

A person from China traveling to a yellow fever–endemic area would usually receive vaccination against yellow fever (*4*). Persons such as our patient, who cannot or should not receive vaccination for yellow fever, should be monitored closely. As of April 2, 2016, a total of 9 imported cases of yellow fever were reported in China: 4 cases in Fujian Province, 4 cases in Beijing, and 1 case in Shanghai. All 9 cases occurred in travelers returning to China from Angola; no local cases have been reported.

The mosquito density is low in Shanghai, and the temperature typically is low in March, suggesting that the imported case we describe will probably not result in mosquito-borne transmission. However, in the upcoming summer, the risk for onward transmission of travel-associated yellow fever in China will warrant increased vigilance. To help prevent the importation and potential spread of yellow fever in China, the Chinese government now requests proof of vaccination for yellow fever from persons traveling to China from Angola.
